# Processing Stage-Induced Formation of Advanced Glycation End Products in Cooked Sausages with the Addition of Spices

**DOI:** 10.3390/foods12203788

**Published:** 2023-10-16

**Authors:** Yong Li, Hua Li, Yinchun Zhu, Cuiping Feng, Zhiyong He, Jie Chen, Maomao Zeng

**Affiliations:** 1 College of Food Science and Engineering, Shanxi Agricultural University, Jinzhong 030801, China; yong.li@sxau.edu.cn (Y.L.); hua.li@sxau.edu.cn (H.L.);; 2 State Key Laboratory of Food Science and Resources, Jiangnan University, Wuxi 214122, Chinachenjie@jiangnan.edu.cn (J.C.); 3 International Joint Laboratory on Food Safety, Jiangnan University, Wuxi 214122, China

**Keywords:** cooked sausages, processing stages, advanced glycation end products (AGEs), 1,2-dicarbonyl compounds, lipid oxidation, protein oxidation, spice

## Abstract

This study aims to evaluate the relationship between the four processing stages of cooked sausage preparation (raw, drying, baking, and steaming) and the formation of advanced glycation end products (AGEs), 1,2-dicarbonyl compounds, and lipid and protein oxidation in sausages with spices. Baking and steaming significantly promoted lipid and protein oxidation. The Nε-carboxymethyllysine (CML) content increased from 4.32–4.81 µg/g in raw samples to 10.68–16.20 µg/g in the steamed sausages. Nε-carboxyethyllysine (CEL) concentrations increased by approximately 1.7–3.7 times after steaming. The methylglyoxal concentration increased dramatically after baking and then rapidly decreased in the steaming stage. Chili promoted the formation of CML and CEL. The CEL concentration increased in samples containing garlic, but yellow mustard and garlic slightly reduced CML concentrations in the cooked sausages. The spices decreased the lipid and protein stability of the cooked sausages, increasing malondialdehyde and protein carbonyls. Lipid oxidation and 3-deoxyglucosone positively correlated with CML and CEL levels. Black pepper had no impact on CML when the sausages were baked but remarkably increased the content of both CML and CEL in the steaming stage. Thus, the impact of spices on sausages depends on both the specific spices used and the category of AGEs formed.

## 1. Introduction

Advanced glycation end products (AGEs) are complex compounds primarily formed via the Maillard reaction, which involves the reaction between free amino acids, peptides, or proteins in food and reactive carbonyls in reducing sugars [1]. Meat and meat products, which are high in protein and fat, serve as important sources of AGEs [1,2]. Moreover, AGEs can also be produced via lipid oxidation during meat processing and storage. 1,2-Dicarbonyl compounds formed via the Maillard reaction and lipid oxidation include methylglyoxal (MGO), glyoxal (GO), and 3-deoxyglucosone (3-DG), which react directly with protein and amino acids to form AGEs [3]. Nε-carboxymethyllysine (CML) and Nε-carboxyethyllysine (CEL) have been extensively used as indicators to detect AGEs in processed meat products [4,5,6]. Dietary AGEs can accumulate in the body, causing oxidative stress and inflammation, resulting in chronic metabolic illnesses, such as diabetes, kidney disease, and neurodegenerative disease [1,7,8].

The formation of CML and CEL is significantly affected by the processing conditions. An increase in the heating temperature of sausages from 90 °C to 130 °C can result in a two- to three-fold increase in the contents of CML and CEL [9]. A survey conducted by Yu et al. [10] revealed that CML and CEL levels in canned meat products sterilized at 121 °C for 20 min were higher (4.6–25 µg/g and 5.5–106 µg/g, respectively) than those in meat products subjected to pasteurization (CML,13.3–40.6 µg/g and CEL, 6.3–21.4 µg/g). Lipid and protein oxidation occurred during the processing and storage of meat products, resulting in undesirable changes in color, taste, and nutritional value [11]. The process of lipid oxidation produces hydroperoxides and other primary oxidation products [9,12]. Protein oxidation during thermal processing might also result in various physicochemical changes in the proteins, including amino acid degradation, protein aggregation, and lower protein digestibility [12]. Protein carbonylation is commonly recognized as the principal outcome of protein oxidation caused by free radicals [12]. Oxidation-induced chemical changes in meat proteins also include the loss of tryptophan fluorescence [12,13]. Moreover, protein oxidation and Maillard reaction play important roles in generating CML and CEL in meat products [14]. The addition to this, food additives might have an impact on these reactions.

Spices are a good source of polyphenols and are commonly regarded as potential inhibitors of Maillard reaction-related products [15,16,17]. For example, garlic, black pepper, and chili inhibited the formation of totally free heterocyclic amines in cooked sausage [16]. Garlic and onion powder effectively decreased lipid oxidation in fresh pork belly [17]. However, distinctions between the study methods used have been noted, such as directly adding spices or their extracts. Additionally, various food and model systems were used to determine the effect of spices on Maillard reaction products [15]. Some spices (ginger and Sichuan pepper), however, have been found to promote the generation of some other Maillard reaction products, such as heterocyclic amines in processed meat [16]. These controversial findings of the impact of spices and antioxidants on the formation of harmful Maillard reaction products might be regarded as an important topic of further investigation. Garlic, black pepper, chili, ginger, and other spices are frequently added to improve the flavor of cooked sausages. However, it remains unclear whether spices have a promoting or lowering effect on AGE generation at different stages of cooked sausage processing. Therefore, this study focused on commonly used spices, such as black pepper, chili pepper, yellow mustard, and garlic powder, as additives to investigate their effects on AGEs, lipids, and protein oxidation during the processing stages (raw, drying, baking, and steaming) of the cooked sausages.

## 2. Materials and Methods

### 2.1. Reagents and Standards

The standards CML, CEL, Nε-(carboxymethyl)lysine-d4 (CML-d4), and Nε-(carboxyethyl)lysine-d4 (CEL-d4) were procured from Toronto Research Chemicals, Inc. (Toronto, ON; Canada). The chemicals o-phenylenediamine (OPD) (99.5%), MGO, and 3-DG were obtained from Sigma (Darmstadt; Germany). Methanol and acetonitrile of chromatographic grade were supplied by Tedia Company, USA. The rest of the analytical reagents were purchased from Sinopharm Chemical Reagent Co., China. Furthermore, pork, salt, food additives, and spices were purchased from a local supermarket located in Jinzhong, China.

### 2.2. Preparation of Sausages

Fresh pork hind leg was minced at 10 °C in a mincer fitted with an adjustable plate with a hole diameter of 4.5 mm. Five types of sausages were prepared. Sausages without spices were used as the control, whereas samples containing black pepper powder (0.5%), chili powder (0.5%), yellow mustard powder (0.5%), and garlic powder (0.5%) were the spiced sausages. The ratio of the spices to be incorporated into the sausages was based on previous studies and the quantity of spices used in sausage production was reported in the literature [15,17,18]. The minced meat was mixed with salt, and their composition was as follows: pork hind leg (62%), pork back fat (28%), ice (10%), NaCl (1.47%), composite phosphate (0.23%), sodium ascorbate (0.05%), and NaNO_2_ (0.01%). Each type of meat was divided into three portions of about 300 g each. The meat batter was refrigerated for 4 h at 4 °C and then stuffed into collagen casings with a diameter of 18 mm using a manual sausage stuffing machine (Linyi Best Food Machinery Co., LTD, Linyi; China); this was then cut into segments approximately 15 cm in length. The processing of each type of cooked sausages consisted of four stages: Stage 1 (raw), Stage 2 (drying, 10 min at 50 °C), Stage 3 (baking, 20 min at 70 °C), and Stage 4 (steaming, 10 min at 100 °C). Depending on the processing stages applied, each type of sausage was divided into four different groups: raw, dried, baked, and steamed sausages. Each treatment was carried out in triplicate. Raw sausages were frozen (−20 °C) after analyzing the color and texture profile. After each stage of processing (drying, baking, and steaming), the samples were cooled to 25 °C and kept at 4 °C for physicochemical analysis; the remaining samples were stored at −20 °C until further analysis.

### 2.3. Physicochemical Analysis

#### 2.3.1. Determination of Cooking Loss and Moisture Content

The moisture content was determined by heating the samples to a constant weight at 105 °C. The cooking loss of sausages was assessed by calculating the percentage weight difference between the raw and processed samples at each stage using the following formula: cooking loss (%) = (final weight/initial weight) × 100.

#### 2.3.2. Texture Profile Analysis (TPA)

The TPA was measured using a TMS-PRO Food Texture Analyzer (Food Technology Corporation, Sterling, VA, USA) coupled with a 100 N load cell and a cylindrical probe (diameter 12.7 mm). After removing the pith, the sausages were divided into 2 cm-long cylindrical pieces for analysis. The test parameters of the texture analyzer were as follows: time interval between two compression tests, 3 s; initial maximum force, 0.4 N; vertical distance at which the probe was elevated from the surface of the sample, 10 mm; extrusion distance, 1/2 the thickness of the samples; test and posttest speed were set as 60 mm/min and 150 mm/min, respectively.

#### 2.3.3. Color

The color of the sausage was measured using a KONICA MINOLTA CM-5 spectrophotometer (Konica Minolta Sensing Americas, Inc., Tokyo, Japan). Before conducting measurements, it was essential to calibrate the colorimeter by using a standard whiteboard. The color parameters were lightness (L*), redness (a*), and yellowness (b*). The L* value means whiteness, with higher values indicating a brighter hue. The a* value represents the presence of red or green tones, with positive values representing red and negative values suggesting green. Similarly, the b* value indicates the presence of yellow or blue tones, with positive values indicating yellow and negative values suggesting blue.

### 2.4. Lipid and Protein Oxidation Indicators

#### 2.4.1. Lipid Oxidation

The lipid oxidation during processing was evaluated using the 2-thiobarbituric acid (TBARS) method, as described by Utrera and Estévez [19]. The absorbance measurement of the supernatant was conducted at 532 nm and 600 nm. The TBARS values were quantified and reported in milligrams of malondialdehyde (MDA) per kilogram of dry weight.

#### 2.4.2. Tryptophan Fluorescence

Tryptophan fluorescence was quantified following the methodology of Gan et al. [20] with some modifications. One gram of each sample was homogenized with six-fold 100 mM sodium phosphate buffer (PBS) at pH 6.5, which included 0.6 M NaCl and 8 M urea. After centrifuging the homogenates at 5000× *g* for 5 min, the supernatants were diluted with PBS for further analysis. The fluorescence intensity was measured using emission spectra at 338 nm and excitation wavelength at 283 nm, with 10 nm slit widths.

#### 2.4.3. Protein Carbonyls

The quantification of protein oxidation via the determination of the total carbonyl content was conducted using dinitrophenylhydrazine (DNPH) derivatization and outlined in the procedure described by Cando et al. [21] and Li et al. [22]. The absorbance values at 280 and 370 nm were determined using the SpectraMax 190 absorbance microplate reader (Molecular Devices, LLC., San Jose, CA, USA). The content of the carbonyl groups was reported in terms of nanomoles of carbonyl per milligram of protein (nmol/mg protein).

### 2.5. Measurement of AGEs

The samples (150 mg) were defatted using n-hexane (5 mL). The defatted powder was reduced overnight at 4 °C in borate buffer (0.2 M, pH 9.2, 0.5 mL) and sodium borohydride (1 M, 0.5 mL). The sample was then hydrolyzed in an oven for 24 h at 110 °C with 4 mL of 6 M HCl. After the hydrolysates were concentrated to a volume of 25 mL and filtered, 2 mL of the solutions were nitrogen blow-dried, and the dried hydrolysates were reconstituted in distilled water (2 mL) spiked with CML-d4 and CEL-d4 internal standards (150 μL, 1 μg/mL). Solid phase extraction was completed using Waters MCX cartridges (60 mg, 3 mL), which were activated using 3 mL each of methanol, ultrapure water, and 0.1 M HCl. The cartridges were then filled with 2 mL of the samples and washed with 3 mL 0.1 M HCl followed by 3 mL of water. The AGEs were then eluted with 4 mL of NH_4_OH/methanol (*v*/*v* = 5/95). The eluate was dried with nitrogen at 50 °C, reconstituted in 300 μL of water, and filtered through a 0.22-μm membrane for HPLC–MS/MS analysis.

The samples (5 μL) were injected into a Waters HPLC equipped with an X-Bridge C18 column (2.1 × 100 mm, 3.5 μm). The HPLC system was equipped with a Quattro Micro triple-quadrupole mass spectrometer. The eluents were 5 mM nonafluoropentanoic acid (A) and acetonitrile (B). The B concentration was varied using a gradient program, ranging from 5% (0.1 min) to 60% (5 min), holding at 100% (7–9 min) and 5% (10–15 min). The flow rate was set at 0.3 mL/min, while the column temperature was 35 °C.

### 2.6. Measurement of 1,2-Dicarbonyl Compounds

MGO and 3-DG were determined, which were derivatized with OPD. To perform derivatization, 200 µL of the extract was mixed with 100 µL of 0.1 mM 2,3-hexanedione internal standard and 200 µL of 5 mM OPD. Derivation was performed at 4 °C for 12 h. The quinoxaline derivatives of 3-DG and MGO were determined by using the Waters UPLC-MS/MS system (Waters, Milford, MA, USA). The separation was conducted on a C18 column (2.1 × 100 mm, 3.5 μm; Waters X-Bridge) with a flow rate of 0.3 mL/min at 35 °C and with 5 µL of the samples. The mobile phase was (A) acetonitrile and (B) 1% formic acid. The gradient elution condition was as follows: A: 10%, 0–0.1 min; 20%,1 min; 70%, 6 min; 100%, 6.3–7.5 min; and 10%, 8–10 min. Positive ESI ionization and MRM scan modes were used. The parent ion and daughter ion were as follows: *m*/*z* 145 → 77 for MGO (cone, 35 v; collision, 25 v), *m*/*z* 235 → 199 for 3-DG (cone, 30 v; collision, 12 v), and *m*/*z* 187 → 77 for 2,3-hexanedione (cone, 32 v; collision, 35 v).

### 2.7. Statistical Analyses

The experiments were performed in triplicate. Analysis of variance (ANOVA) (physicochemical, textural profiles, protein oxidation, 1,2-dicarbonyl compounds, and AGEs) was conducted by using linear models and a completely randomized design procedure of Statistix (version 9). Two-way ANOVA was used to evaluate the interaction between processing stages and spices on AGEs. All-pairwise comparisons and the least significant differences (LSD) were used to determine the significance of different treatments (*p* < 0.05). The results were reported as means values and standard deviation. For the protein oxidation indicators, MGO, 3-DG, CML, and CEL, principal component analysis (PCA), and correlation were implemented by using SIMICA-14.1 and Origin pro, respectively.

## 3. Results and Discussion

### 3.1. Physicochemical Analysis

#### 3.1.1. Changes in Cooking Loss and Moisture Content

Cooking loss increased steadily throughout the processing stages (*p* < 0.05) (Table 1). The control sausage had eight times higher cooking loss in the steamed stage than in the dried stage (9.84 ± 2.07% vs. 1.23 ± 0.27%). Additionally, the moisture content decreased from 52.78% of the raw sausage to 42.67% of the final samples, a decrease of approximately 19%. Sausages treated with other spices maintained a moisture content between 45 and 48% after the steaming process, which is slightly higher than that of the control group (43%); these results are consistent with those of Yang et al. [16]. Myofibrillar protein denaturation and protein-induced oxidative damage during high-temperature processing can result in muscle fiber contraction, cell membrane deterioration, decrease in protein water-holding capacity, and migration of water and water-soluble components. This led to a decrease in moisture content and an increase in cooking loss [11]. Cooking loss of the sausages treated with spices at the steaming stage was considerably increased (*p* < 0.05) and nearly doubled compared to the control group (Table 1). This might be because spices change the osmotic pressure of the sausage system, influencing the moisture content and cooking loss. Spices might disrupt the force between the myofibrils, making it easier to lose water during the steaming process.

#### 3.1.2. Changes in Color Parameters and TPA

The L* value decreased substantially during baking (*p* < 0.05); however, it increased considerably following steaming. Moreover, compared to the control group, the addition of black pepper, chili, and garlic dramatically decreased the L* value of steamed sausages (Table 1). As for the a* values, it was observed that with an increase in the intensity of thermal processing, the a* values of the control samples, chili, and yellow mustard powder treatments were significantly reduced (*p* < 0.05). However, in the case of sausages with garlic treatment, the a* value increased significantly (*p* < 0.05), ranging from 0.69 to 2.33 after the steaming process. These results confirmed that the color of sausages changed during different processing stages, mainly due to the loss of red color (in contrast to the garlic treatment samples). It was also observed that the a* values of the chili pepper group at different processing stages ranged from 6.81 to 9.16, significantly higher than those of the control group. This suggests that adding red chili powder plays a crucial role in increasing the a* value of those sausages. It was found that the impact of the drying and baking stages on the b* values was relatively small when compared with the raw samples. However, during the steaming step, an increase was observed for all treatments (Table 1). Moreover, the b* values of the steamed sausages that were processed with spices (black pepper, 16.99 ± 0.80; chili, 23.87 ± 1.05; yellow mustard, 18.11 ± 1.13; garlic, 18.85 ± 1.01) were significantly higher than those of the control group (15.27 ± 1.75).

The results of the texture analysis showed that the hardness of the control group increased from 3.40 ± 0.67 N to 27.32 ± 2.98 N (*p* < 0.05) due to the different processing stages. Similar changes in the TPA indicators were observed in the sausages processed with other spices at different stages (data not presented). Yellow mustard and control sample were lower than other spices for gumminess and chewiness. These spices might have a greater influence on the force between the myofibrils. No significant difference (*p* > 0.05) was observed between the control and each sausage with spice treatment (except Garlic) in terms of adhesion, cohesiveness, and springiness (Table 2). These results are consistent with those of the study of Yang et al. [16], which focused on smoked and cooked sausages treated with ginger, Sichuan pepper, black pepper, etc.

### 3.2. Lipids and Protein Oxidation in Sausages during the Different Processing Stages

#### 3.2.1. Lipid Oxidation

The quantification of lipid and protein oxidation products in the sausages at different processing stages was performed (Table 3). The TBARS method was employed to assess the levels of MDA. According to Table 3, no significant changes in TBARS were observed (*p* > 0.05) for all sausages in the raw and dried stages of processing. However, the TBARS levels increased considerably after steaming (2.11 ± 0028–3.34 ± 0.059 mg MDA/kg), resulting in a 7.6–12.2-fold increase compared to raw samples. The TBARS value was expected to increase with the extent of the thermal condition, as lipid oxidation is easily impacted by heat treatment, which was accelerated at high temperatures [23]. The results of the drying stages in the present experiments were in line with results from a previous study indicating no differences (*p* > 0.05) in sheep sausages treated with Origanum vulgare extract [24]. Additionally, Cando et al. [21] found no significant differences in the TBARS values between the cooked meat products with and without antioxidants or with greater amounts of natural antioxidants. On the contrary, in the present study, spices significantly promoted lipid oxidation in the sausages during the steaming process (Table 3). Bao et al. [23] also discovered that adding 0.5% and 1.0% black pepper to fried tilapia fillets increased the TBARS levels. It was reported that adding 0.5% garlic to beef patties, followed by vacuum packaging and heating in a 70 °C water bath for 70 min, greatly increased the formation of MDA [25]. However, the results of the present study are inconsistent with those of Aguirrezábal et al. [26] who discovered that the addition of garlic (1%) to dry fermented sausage, chorizo, significantly reduced the TBARS values during the ripening period. This may be attributed to the variances in sausage type, processing procedures, and amounts of spices used. Therefore, the impact of spices on lipid oxidation was complex, although garlic and other spices as well as their extracts had varying degrees of antioxidant properties [27,28].

#### 3.2.2. Changes in Tryptophan Fluorescence Intensity

The decrease in tryptophan fluorescence has been attributed to the oxidative degradation and conversion of tryptophan into other compounds. Table 3 shows that the tryptophan fluorescence intensity in the sausage significantly decreased with the progression of thermal processing in all groups except for the samples with yellow mustard. The range of the reduced rate of tryptophan fluorescence intensity in the control samples was between 26% and 34%. The amino acid residue of tryptophan is located in the inner core of natural proteins. Thus, the decline in fluorescence intensity can be attributed to both the direct oxidative destruction of tryptophan and the unfolding of proteins, which subsequently exposes tryptophan to the surrounding solvent [19,22]. Compared with the raw sausages, the tryptophan fluorescence intensity in the steamed samples of the control, black pepper, chili, and garlic-added groups decreased by 33.4%, 26.0%, 32.9%, and 34.4%, respectively (Table 3). During the steamed stage, no significant difference in the tryptophan fluorescence intensity was observed between the control, chili, and garlic treatments.

#### 3.2.3. Changes in Protein Carbonyls

Table 3 shows the changes in the total carbonyl content in different processing stages. The protein carbonyl content was observed to have an increasing trend after steaming, which was opposite to the changes in the tryptophan fluorescence intensity (Table 3). The results are in line with those of the study of Sun et al. [29] who investigated the carbonylation phenomenon in several muscle proteins during the manufacturing of fermented Guangdong sausages and observed that the fermentation and oven-drying processes of sausages resulted in a progressive and substantial elevation in carbonyl levels within the muscle proteins. The overall amount of protein carbonyls and particular indices of protein oxidation (α-aminoadipic and γ-glutamic semialdehydes) in lamb loins increased gradually during the sous vide cooking process, exceeding the levels detected in uncooked samples [30]. High temperatures during the thermal process damaged the cell compartments, resulting in the release of free catalytic iron and the generation of hydrogen peroxide, which promoted protein carbonylation [30]. Furthermore, the interplay between proteins and reactive oxygen species might facilitate the formation of carbonyls during several processing stages.

During the steaming stage, the black pepper, garlic, and chili groups of sausages showed varying degrees of carbonyl content increase, although not all changes were statistically significant (Table 3). Black pepper, for example, had a much higher protein carbonyl content (9.37 nmol/mg protein) than other samples (4.49–7.69 nmol/mg protein). Similar results have been reported by Bao et al. [23] who reported that the fried tilapia fillets with 1.0% and 1.5% black pepper had more protein carbonyls than the control samples. Although the addition of black pepper to sausages increased protein oxidation, this was not the case with yellow mustard (Table 3). Furthermore, similar findings have been found in cases where spices used as antioxidants may block the formation of harmful Maillard reaction products [16].

### 3.3. Changes in AGE Profiles

The content of CML and CEL exhibited little changes at the drying stage. Nevertheless, in the baking and steaming stages, the CML and CEL concentrations in sausages increased rapidly (Table 4). These findings are consistent with those of earlier studies that have shown that the CML and CEL levels moderately increased at lower temperatures and rapidly increased when the temperatures approached 100 °C [9,31]. The CML content in the sausages increased from 4.32–4.81 µg/g in Stage 1 (raw) to 10.68–16.2 µg/g in Stage 4 (steaming), indicating an increase of about 2.2–2.9 times (Table 4). As for CEL, its concentration in the raw sample ranged from 4.79 to 7.95 µg/g. However, after baking and steaming, the content increased by approximately 1.7–3.7 times (Table 4). The CML and CEL contents in fermented and cooked sausages were also within a comparable range (from 3.67 to 46.11 µg/g and 5.89 to 52.32 µg/g, respectively) [32]. Meat lipids and protein are prone to oxidation in elevated temperatures (baking and steaming), resulting in protein unfolding, and the exposure of active amino acids, which then participated in the Maillard reaction contributing to the generation of AGEs [33]. Lu et al. [9] reported that an increase in the heating temperature of sausages from 90 °C to 130 °C resulted in a two- to threefold increase in the contents of CML and CEL. The lipid and protein oxidation caused during the process was correlated with the formation of CML and CEL as reported by Yu et al. [34].

In the sausages treated with spices, the highest CML content in the black pepper group reached 16.22 µg/g, promoting the formation of CML, whereas yellow mustard and garlic slightly reduced the CML concentrations in sausage, with decreasing rates of 16% and 13%, respectively (Table 4). Additionally, black pepper and chili significantly increased the CEL formation. Since the temperatures utilized at each stage of the sausage production varied greatly, the effects of the spices on AGEs were exerted differently at each stage. The addition of powdered spices might disturb the osmotic pressure inside muscle tissue, causing structural injury and enhancing the vulnerability of myofibrils to free radicals and other pro-oxidant factors [23]. This impact may play a dominant role than the antioxidant- and free radical-scavenging capacities of the anti-oxidative compounds present in these spices, accelerating lipid jiaohuoxidation and the Maillard reaction and resulting in the formation of AGEs [3,16,23,28]. The interactions between processing stages and spices were significant (*p* < 0.05) (Table 5). These interactions and their impact on AGEs would thus likely affect product quality.

### 3.4. Changes in 1,2-Dicarbonyl Compounds

During the Maillard reaction, a range of 1,2-dicarbonyl compounds are generated. These include methylglyoxal (MGO) and 3-deoxyglucosone (3-DG). The MGO concentration remained consistent in the raw (0.065 ± 0.0055–0.093 ± 0.013 µg/g) and dried samples but increased dramatically after baking, with an increase of approximately 2.7–4.2 times (Table 6). Subsequently, MGO rapidly decreased to levels comparable to that of the raw samples during the steaming process (0.073–0.12 µg/g). These results are in line with those of Lu et al. [9] who also found that the MGO content in cooked sausages decreased after 2 h of heating. These findings suggest that the generation of MGO may decrease under the condition of heat, given that 1,2-dicarbonyls serve as intermediates in the Maillard reaction. MGO may degrade under conditions of high temperatures, such as grilling, roasting, and frying [35]. However, the MGO contents in the present study were lower than the results of Yusufoğlu et al. [36] who reported that MGO in Turkish traditional sausage containing fruits ranged from 1.25 to 27.66 µg/g. The main reason was that the sugars in these high-sugar-containing sausages can generate a large number of 1,2-dicarbonyl structures via the Maillard reaction and the caramelization reaction, which causes an increase in MGO content in the sausages. Sausages supplemented with chili, yellow mustard, and garlic showed a substantial reduction in MGO content as compared with the control samples. These effects on MGO might be attributed to the antioxidant activity of certain spices. Furthermore, chili pepper is rich in flavonoids, and its proper incorporation can potentially prevent the Maillard reaction [16]. Both garlic and yellow mustard are rich in sulfur compounds and their derivatives [37]. These chemicals have the potential to directly engage in the Maillard reaction and deplete the glucose precursor of the MGO [16,37].

However, the spices significantly promoted the formation of 3-DG (Table 6). The contents of 3-DG showed a significant increase during the baking and steaming stages. Spices, processing stages, and their interaction had a significant effect on 3-DG (*p* < 0.001 for all of them). When compared with the raw samples, the 3-DG in steamed sausages increased by 1.5–2.8 times (control, 0.029; black pepper, 0.089; chili, 0.29; yellow mustard, 0.057; garlic, 0.12 µg/g), indicating a gradual increase in levels under high temperatures, which might potentially be attributed to the synchronous impact of both the Maillard reaction and lipid oxidation [38]. During the Maillard reaction, the formation of Amadori rearrangement products takes place, which subsequently undergo enolization and dehydration processes, resulting in the generation of reactive dicarbonyl compounds [38], among which 3-DG is one of the most active dicarbonyl compounds readily react with the amino groups in proteins to form a variety of AGE structures in the later phases of the Maillard reaction. Among these AGEs, it has been observed that CML content is the most prevalent [39].

### 3.5. Principal Component Analysis (PCA) and Correlation Analysis

**Figure 1:**
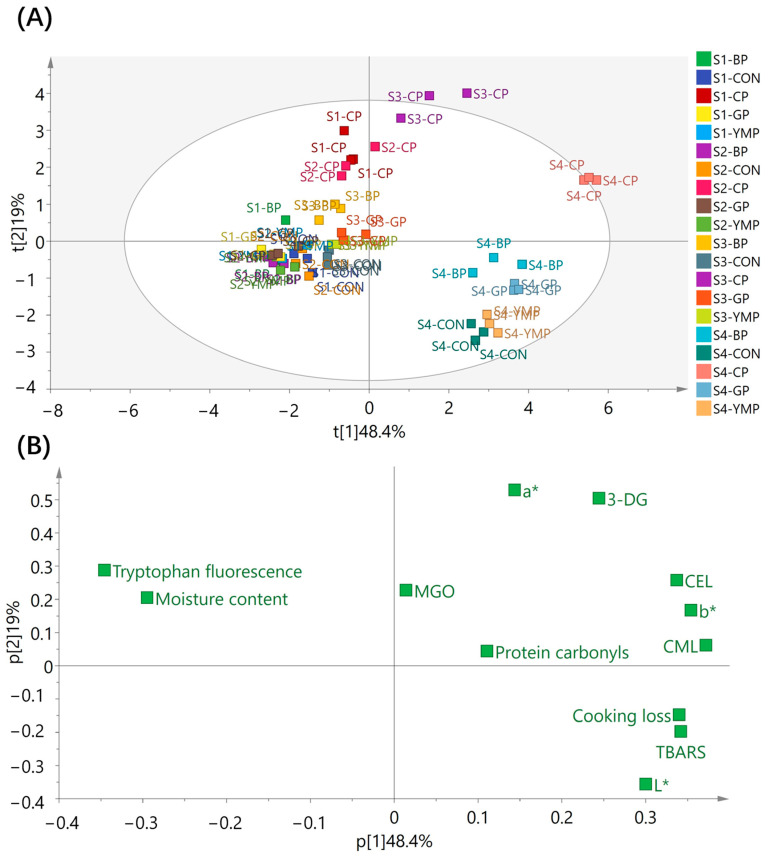
Principal component analysis (score plot, **A**; loading plot, **B**) of physicochemical composition, lipid, and protein oxidation indicators, 1,2-dicarbonyl compounds, and advanced glycation end products of sausages treated with spices and different thermal processing stages. S1–S4, stage 1–stage 4; Stage 1, raw; Stage 2, drying; Stage 3, baking; and Stage 4, steaming. CON, control; BP, black pepper; CP, chili powder; YMP, yellow mustard powder; GP, garlic powder.

PCA was conducted to determine the differences between sausages treated with spices and undergoing the processing stages based on physicochemical composition, lipid and protein oxidation, 1,2-dicarbonyl compounds, and AGE levels. The score plot (Figure 1A) presents the PCA plot for the formation of lipid and protein oxidation and AGEs in the control group at various processing stages. Principal component (PC) 1 has divided these datasets based on the processing stages. Specifically, the samples from stages 1 to 3 (raw, drying, and baking) were all situated on the left side of the plot, whereas the samples from Stage 4 (steaming) were all located on the right, implying that the processing stages greatly influenced the AGEs, 1,2-dicarbonyl compounds, and lipid oxidation profiles. The loading plot in Figure 1B shows that the positions of CML, CEL, 3-DG, TBARS, protein carbonyls, cooking loss, and color (L*, a*, and b*) are comparable with the locations of the steaming samples in the score plot, indicating that the sausages at this processing stage contained more of these products than the sausages at the earlier stages. This finding supported the role of cooking in the development of lipid oxidation and the formation of AGEs. The chili-treated sausages in the baking stage (Stage 2) clustered on the right side of the vertical axis in the score plot, which was similar to the sample points of Stage 4 (Figure 1A), indicating that the addition of chili considerably increased the generation of CML, CEL, MGO, and 3-DG in the sausages during the baking stage.

**Figure 2:**
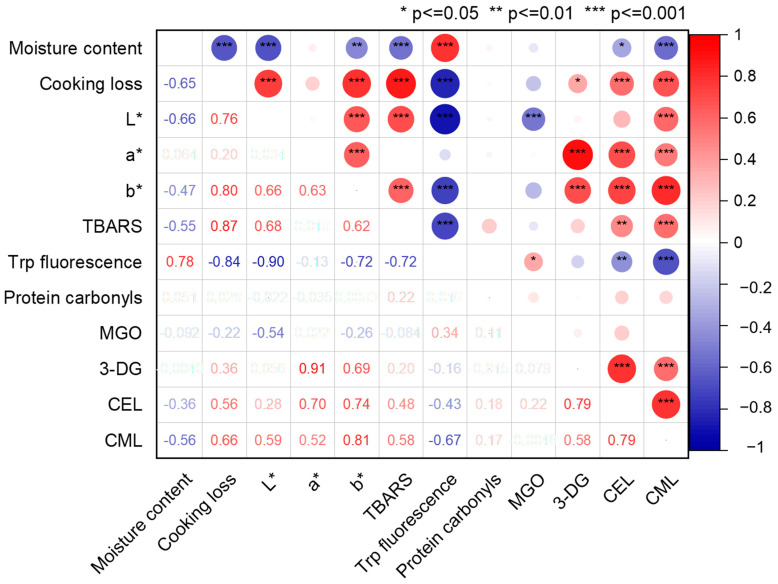
Pearson correlations (r) between the physicochemical composition, lipid and protein oxidation indicators, 1,2-dicarbonyl compounds, and AGEs. * *p* ≤ 0.05, ** *p* ≤ 0.01, and *** *p* ≤ 0.001; MGO, methylglyoxal; 3-DG, 3-deoxyglucosone; CML, Nε-carboxymethyllysine; CEL, Nε-carboxyethyllysine.

Figure 2 shows that the moisture content was negatively correlated with CML and CEL contents (r = −0.56 and r = −0.36, respectively, *p* < 0.05) and positively correlated with cooking loss, suggesting that the sausages with a higher moisture content tend to have lower levels of AGEs. The continuous evaporation of water occurs during thermal processing, therefore enabling its utilization as a medium for both transportation and chemical reactions. The precursors of AGEs migrate to the sausage surface with water during heating. Subsequently, the precursors are subjected to higher temperatures on the sample surface, leading to a continuous formation of AGEs [22]. In the present study, the TBARS and 3-DG values were positively correlated with AGEs (CML: r = 0.58 and r = 0.58, respectively, *p* < 0.05) (CEL: r = 0.48 and r = 0.79, *p* < 0.05). Negative correlations were also observed between Trp fluorescence and AGEs (CML: r = 0.67 and CEL: r = 0.43, *p* < 0.05). As an important active carbonyl compound in the Maillard reaction, 3-DG readily reacts with amino groups in proteins participating in the pathway of AGE formation. Among these AGEs, it has been observed that CML is the most prevalent [36]. Free radicals and hydroperoxides, which are products of lipid oxidation, interact with the sensitive amino acids of proteins, resulting in the unfolding of myosin, exposing a greater number of amino acid residues, including tryptophan and lysine residues to the surrounding environment [11]. These alterations might result in a lower tryptophan fluorescence and more free lysine residues interacting with 1,2-dicarbonyl-intermediates, inducing higher CML and CEL levels.

## 4. Conclusions

Most of the spice powders used impacted the content of AGEs in cooked sausages. However, this effect depended on the type of spices and the stages of thermal processing. The thermal processing stages accelerated the formation of AGEs. Furthermore, the addition of spices in sausage production can potentially lead to an elevation in the contents of CML. This effect is dependent on the specific stages of processing. In the case of black pepper, it had no impact on the formation of CML when sausages were baked; however, it significantly enhanced the concentration of both CML and CEL in the steaming stages. The results of our study also indicate that the impact of spices on sausages depends on the specific type of spices used and the category of AGEs formed. The concentrations of CML and CEL increased when chili was added. The contents of CML were reduced in the cooked sausages with yellow mustard and garlic; however, the CEL concentration increased in the samples containing garlic. The interactions between processing stages and spices significantly impacted AGEs and 1,2-dicarbonyl compounds. Among the four stages of the sausage preparation, baking and steaming significantly promoted lipid and protein oxidation. All spices promoted lipid oxidation of the cooked sausages, whereas black pepper and garlic increased the formation of protein carbonyls. TBARS and 3-DG were significantly and positively correlated with the formation of CML and CEL (*p* < 0.05). Overall, it is important to be mindful of the thermal processing stages and the choice of spices during sausage preparation.

## Figures and Tables

**Table 1:** Effects of processing stages and spices on cooking loss, moisture content, and color of cooked sausages.

		Cooking Loss (%)	Moisture Content (%)	Color Parameters		
				L* (Lightness)	a* (Redness)	b* (Yellowness)
Control	raw	_	52.78 ± 0.26 aC	45.15 ± 4.68 bA	2.41 ± 0.50 aB	12.17 ± 1.19 bB
	drying	1.23 ± 0.27 aB	49.43 ± 0.13 bD	42.14 ± 3.33 bcA	2.01 ± 0.51 aB	10.69 ± 1.69 bB
	baking	4.75 ± 0.84 bB	45.73 ± 0.10 cD	39.19 ± 2.70 cA	1.26 ± 0.43 bB	10.41 ± 1.63 bBC
	steaming	9.84 ± 2.07 cB	42.67 ± 0.11 dE	57.50 ± 2.06 aA	1.30 ± 0.36 bD	15.27 ± 1.75 aD
Black pepper	raw	_	51.96 ± 0.32 aD	40.59 ± 4.72 bB	2.54 ± 0.80 aB	10.48 ± 1.67 bB
	drying	1.37 ± 0.28 aB	51.07 ± 0.26 bB	41.04 ± 3.97 bA	1.29 ± 0.36 bC	7.90 ± 0.73 cC
	baking	5.46 ± 0.72 bB	49.20 ± 0.17 cA	37.60 ± 2.86 bA	1.61 ± 0.3 bB	9.45 ± 1.54 bC
	steaming	12.76 ± 2.56 cB	47.91 ± 0.16 dA	51.47 ± 1.92 aB	2.56 ± 0.28 aB	16.99 ± 0.80 aC
Chili	raw	_	53.36 ± 0.24 aB	41.90 ± 1.93 bAB	9.16 ± 1.38 aA	17.94 ± 2.16 bA
	drying	1.45 ± 0.29 aB	51.73 ± 0.19 bA	42.54 ± 3.51 bA	6.81 ± 0.77 bA	15.16 ± 2.60 cA
	baking	5.36 ± 0.42 bB	47.58 ± 0.12 cC	37.97 ± 2.84 cA	7.08 ± 0.87 bA	16.70 ± 1.81 bcA
	steaming	20.88 ± 2.47 cA	46.95 ± 0.14 dB	52.03 ± 1.27 aB	7.35 ± 0.56 bA	23.87 ± 1.05 aA
Yellow mustard	raw	_	53.16 ± 0.21 aBC	44.03 ± 2.47 bAB	2.96 ± 1.19 aB	12.32 ± 2.14 bB
	drying	1.33 ± 0.33 aB	50.94 ± 0.23 bB	43.02 ± 2.93 bA	1.25 ± 0.43 bCD	10.95 ± 1.31 bcB
	baking	4.78 ± 0.91 bB	49.26 ± 0.10 cA	39.95 ± 2.39 cA	1.41 ± 0.36 bB	9.89 ± 1.47 cC
	steaming	19.53 ± 3.15 cA	45.4 ± 0.14 dD	56.73 ± 1.56 aA	1.96 ± 0.29 bC	18.11 ± 1.13 aBC
Garlic	raw	_	55.29 ± 0.21 aA	42.06 ± 3.57 bAB	0.69 ± 0.24 bC	11.48 ± 1.85 bB
	drying	1.82 ± 0.38 aA	50.06 ± 0.32 bC	40.59 ± 2.34 bA	0.73 ± 0.14 bD	10.37 ± 1.23 bB
	baking	6.35 ± 0.45 bA	48.78 ± 0.17 cB	39.76 ± 3.03 bA	0.70 ± 0.20 bC	11.79 ± 0.72 bB
	steaming	21.36 ± 1.87 cA	45.93 ± 0.15 dC	51.90 ± 1.79 aB	2.33 ± 0.39 aBC	18.85 ± 1.01 aB

a–d Values with different letters within a column of each processing stage are significantly different (*p* < 0.05); A–E Values with different letters within a column of different spices with the same processing stage are significantly different (*p* < 0.05).

**Table 2:** Effects of spices on steamed sausages through texture profile analysis (TPA).

Steamed	Hardness	Adhesiveness	Cohesiveness	Springiness	Gumminess	Chewiness
Sausage	N	N.mm	Ratio	mm	N	mJ
Control	31.4 ± 2.98 BC	0.11 ± 0.02 A	0.22 ± 0.01 A	9.4 ± 0.5 AB	6.8 ± 0.57 BC	64.05 ± 7.54 AB
Black pepper	34.32 ± 3.2 AB	0.1 ± 0.02 A	0.21 ± 0.03 A	8.95 ± 1.31 B	7.36 ± 1.31 ABC	70.09 ± 15.77 AB
Chili	34.67 ± 2.64 A	0.1 ± 0.02 A	0.22 ± 0.02 A	9.62 ± 0.47 A	7.66 ± 1.04 AB	73.79 ± 11.93 A
Yellow mustard	28.45 ± 1.99 C	0.11 ± 0.03 A	0.22 ± 0.02 A	9.44 ± 0.22 AB	6.23 ± 0.94 C	58.9 ± 10.33 B
Garlic	36.11 ± 2.67 A	0.09 ± 0.02 A	0.22 ± 0.02 A	9.63 ± 0.49 A	7.89 ± 0.87 A	76.27 ± 11.85 A

Capital letters (A–C) indicate significant differences among spices within the same column (*p* < 0.05).

**Table 3:** Concentrations of malondialdehyde, tryptophan fluorescence, and protein carbonyls in thesausages during the different processing stages with the addition of spices.

Spices	Stages	TBARS (mg MDA/kg)	Tryptophan Fluorescence Intensity	Total Carbonyl Content (nmol/mg Protein)
Control	Raw	0.28 ± 0.0062 bB	993.00 ± 15.56 bB	6.12 ± 0.34 aA
	Drying	0.24 ± 0.035 bAB	1058.50 ± 31.82 aB	6.40 ± 0.96 aA
	Baking	0.25 ± 0.031 bD	989.50 ± 16.26 bB	6.29 ± 0.10 aAB
	Steaming	2.11 ± 0.028 aE	661.50 ± 4.95 cB	6.45 ± 0.35 aB
Black pepper	Raw	0.27 ± 0.0078 cBC	1086.00 ± 32.53 aA	5.04 ± 2.04 cA
	Drying	0.25 ± 0.011 cA	1061.50 ± 95.46 aB	6.09 ± 1.93 bcA
	Baking	1.71 ± 0.063 bC	1196.50 ± 4.95 aA	8.22 ± 1.25 abA
	Steaming	2.85 ± 0.038 aD	803.50 ± 2.12 bA	9.37 ± 1.04 aA
Chili	Raw	0.26 ± 0.0085 bC	1090.00 ± 62.23 aA	5.78 ± 0.91 aA
	Drying	0.23 ± 0.0077 cAB	1118.00 ± 26.87 aAB	6.52 ± 0.30 aA
	Baking	0.28 ± 0.016 bD	1075.00 ± 131.52 aAB	5.57 ± 1.04 aB
	Steaming	3.22 ± 0.033 aB	731.00 ± 4.24 bAB	6.71 ± 0.15 aB
Yellow mustard	Raw	0.27 ± 0.0094 cBC	1063.50 ± 0.71 aAB	4.65 ± 0.66 bcA
	Drying	0.21 ± 0.018 cB	1112.50 ± 27.58 aAB	6.19 ± 1.30 abA
	Baking	2.29 ± 0.042 bA	1096.50 ± 33.23 aAB	7.49 ± 0.79 aA
	Steaming	3.06 ± 0.11 aC	703.50 ± 20.51 bAB	4.80 ± 1.066 cC
Garlic	Raw	0.29 ± 0.0027 cA	1087.00 ± 5.66 aA	5.89 ± 0.79 aA
	Drying	0.22 ± 0.0085 dAB	1232.50 ± 10.61 aA	5.92 ± 2.17 aA
	Baking	1.98 ± 0.069 bB	1127.00 ± 111.72 aAB	6.62 ± 0.77 aAB
	Steaming	3.34 ± 0.059 aA	710.5 ± 30.41 bAB	7.85 ± 0.91 aB
Spices		<0.001	0.324	<0.001
Stages		<0.001	0.004	<0.001
Spices * Stages		<0.001	0.003	<0.001

“Spices * Stages” means the interactions between processing stages and spices. Lowercase letters (a–d) indicate significant differences in each processing stage (*p* < 0.05), whereas capital letters (A–E) indicate significant differences among spices within the same processing stage (*p* < 0.05).

**Table 4:** Changes in advanced glycation end products: CML and CEL in cooked sausages with the addition of spices during the different thermal processing stages.

Spices	Stages	CML (μg/g)	CEL (μg/g)
Control	Raw	4.32 ± 0.19 cA	7.74 ± 1.56 bA
	Drying	5.90 ± 0.42 bBC	6.66 ± 1.93 bB
	Baking	6.49 ± 0.35 bB	9.35 ± 1.70 bB
	Steaming	12.69 ± 0.28 aBC	13.61 ± 1.74 aC
Black pepper	Raw	4.26 ± 0.57 bA	7.95 ± 1.76 bA
	Drying	4.93 ± 0.59 bBC	5.38 ± 1.19 bB
	Baking	6.89 ± 0.48 bB	15.26 ± 4.23 aB
	Steaming	16.22 ± 3.26 aA	18.56 ± 3.87 aB
Chili	Raw	4.81 ± 1.42 cA	7.34 ± 2.36 bAB
	Drying	8.15 ± 1.38 bA	13.44 ± 3.47 bA
	Baking	13.96 ± 1.35 aA	25.25 ± 7.87 aA
	Steaming	14.82 ± 1.17 aAB	27.24 ± 2.25 aA
Yellow mustard	Raw	4.80 ± 0.22 cA	4.79 ± 0.77 cB
	Drying	4.25 ± 0.91 cC	5.92 ± 0.92 cB
	Baking	6.39 ± 0.32 bB	11.18 ± 1.32 bB
	Steaming	10.68 ± 0.57 aC	13.42 ± 0.56 aC
Garlic	Raw	4.42 ± 0.40 cA	6.42 ± 0.94 bAB
	Drying	6.24 ± 1.37 bB	7.11 ± 1.98 bB
	Baking	7.22 ± 0.21 bB	12.95 ± 3.88 aB
	Steaming	11.07 ± 0.94 aC	16.65 ± 2.17 aBC

Lowercase letters (a–c) indicate significant differences in each processing stage (*p* < 0.05), whereas capital letters (A–C) indicate significant differences among spices within the same processing stage (*p* < 0.05).

**Table 5:** Type III model ANOVA of CML and CEL.

	CML					CEL				
Source	SS	DF	Mean Square	F	Pr > F	SS	DF	Mean Square	F	Pr > F
Model ^a^	860.186 ^a^	19	45.273	38.696	<0.001	2310.857 ^a^	19	121.624	15.020	<0.001
Stages ^b^	638.023	3	212.674	181.779	<0.001	1311.770	3	437.257	54.000	<0.001
Spices ^c^	108.955	4	27.239	23.282	<0.001	701.027	4	175.257	21.644	<0.001
Stages * Spices	113.209	12	9.434	8.064	<0.001	298.060	12	24.838	3.067	0.004
Error	46.798	40	1.170			323.893	40	8.097		
Corrected total	906.985	59				2634.750	59			

^a^. CML: R^2^ = 0.948; CEL: R^2^ = 0.877; ^b^. Processing stages included raw, drying, baking, and steaming. ^c^. Spices included control, black pepper, yellow mustard, chili, and garlic.

**Table 6:** Changes in the content of methylglyoxal (MGO) and 3-deoxyglucosone(3-DG) in sausages with the addition of spices during different thermal processing stages.

Spices	Stages	MGO (μg/g)	3-DG (μg/g)
Control	Raw	0.093 ± 0.013 bA	0.020 ± 0.00034 cC
	Drying	0.091 ± 0.025 bA	0.022 ± 0.0034 bcC
	Baking	0.25 ± 0.031 aB	0.030 ± 0.0035 aE
	Steaming	0.11 ± 0.022 bA	0.029 ± 0.0057 abE
Black pepper	Raw	0.065 ± 0.0055 cB	0.046 ± 0.0099 cB
	Drying	0.071 ± 0.0053 cAB	0.057 ± 0.0038 bcB
	Baking	0.27 ± 0.017 aB	0.070 ± 0.012 abC
	Steaming	0.12 ± 0.0052 bA	0.086 ± 0.0042 aC
Chili	Raw	0.080 ± 0.015 bAB	0.17 ± 0.0080 cA
	Drying	0.069 ± 0.0070 bAB	0.17 ± 0.015 cA
	Baking	0.33 ± 0.055 aA	0.25 ± 0.0072 bA
	Steaming	0.073 ± 0.011 bB	0.29 ± 0.014 aA
Yellow mustard	Raw	0.085 ± 0.0023 bA	0.026 ± 0.0096 bC
	Drying	0.082 ± 0.010 bAB	0.028 ± 0.0020 bC
	Baking	0.28 ± 0.0073 aB	0.051 ± 0.0058 aD
	Steaming	0.089 ± 0.0085 bB	0.056 ± 0.0018 aD
Garlic	Raw	0.078 ± 0.0051 bAB	0.044 ± 0.0055 cB
	Drying	0.065 ± 0.0047 cB	0.052 ± 0.0069 cB
	Baking	0.25 ± 0.0074 aB	0.089 ± 0.0078 bB
	Steaming	0.081 ± 0.0066 bB	0.12 ± 0.0056 aB
Spices		0.076	<0.001
Stages		<0.001	<0.001
Spices * Stages		<0.001	<0.001

Lowercase letters (a–c) indicate significant differences in each processing stage (*p* < 0.05), whereas capital letters (A–E) indicate significant differences among spices within the same processing stage (*p* < 0.05).

## Data Availability

The data presented in this study are available on request from the corresponding author.
